# Zoonosis–Why we should reconsider “What's in a name?”

**DOI:** 10.3389/fpubh.2023.1133330

**Published:** 2023-02-13

**Authors:** Balbir B. Singh, Michael P. Ward, Polychronis Kostoulas, Navneet K. Dhand

**Affiliations:** ^1^Centre for One Health, Guru Angad Dev Veterinary and Animal Sciences University, Ludhiana, Punjab, India; ^2^Sydney School of Veterinary Science, The University of Sydney, Camden, NSW, Australia; ^3^Department of Veterinary Microbiology, University of Saskatchewan, Saskatoon, SK, Canada; ^4^Faculty of Public Health, University of Thessaly, Volos, Greece

**Keywords:** *zoonosis*, history, etymologia, classification, definition

## 1. Introduction

In 1855, Rudolph Virchow (1821–1902) used the word *zoonosis* for the first time in his famous “Handbook of Communicable Diseases” (1). The description of the word *zoonoses* or *zoonosis* is etymologically varied, although there seems to be a consensus in the published literature that *zoonosis* is a combination of two or three Greek words. The first version is that *zoonosis* is derived from two Greek words “ζῷ*oν*” (zóon—animal or living species) and “νóσoζ” (nósos—disease or unwell) (2). The second version uses the word “noson” in place of “nosos” but has a similar meaning (3, 4). The third version is that *zoonosis* is a combination of the words “zoo” (relating to animals or more generally to life or living things), “noso” (a person who studies disease) and “osis” (referring to a disease) (5). It is also important to note that the terms *anthroponosis* (plural -*es*; when the source is an infectious human; interhuman transfer is typical) and *sapronosis* (plural -*es*; the source is an abiotic substrate, non-living environment; interhuman transfer is exceptional) were also common for non-zoonotic human infections (6). Note the common usage of the suffix “nosis” after the stem of all the three terminologies (anthroponosis, zoonosis and sapronosis).

Independent literature on the roots and origins of medical terms describe the meaning of these Greek words as follows: zóon (‘ζῷ*oν*”)—animal or living thing; nósos (“νóσoζ”)—disease, plague, anguish; osis—a suffix meaning a condition, process, activity (7). Therefore, it is highly likely that “zoon” and “nosos” are the most appropriate words in combination to form the word “zoonosis”. The above suggests the etymologic meaning of *zoonosis* to be either *diseases of animals* or *the study of diseases of animals*. The usage of the words “zoon” and “nosos” is not limited to Greek. As human life was greatly influenced by animals and languages are believed to have a common ancestor, the parallel existence of these words in other languages is expected. For example, the words “Joon” (meaning a living species, or much more commonly an animal species) and “naasaaz” (meaning unwell or unrhythmic) exist in some of the Indo-Aryan languages.

## 2. The standard definition (1978) and the stalemate thereof

The historic legacy and diversity of the word *zoonosis* was also implicitly embedded in its definition when it was defined by the Joint WHO/FAO Expert Group on Zoonoses. In their first report in 1950 The Expert Group defined zoonosis as “those diseases which are naturally transmitted between vertebrate animals and man” (8).

In their 1958 meeting (second report), the Joint WHO/FAO Expert Group on Zoonoses noted that terms such as anthropo-zoonoses (diseases transmitted from animals to man) and zoo-anthroponoses (diseases transmitted from man to animals) had been proposed in the published literature (9). However, it was the committee's opinion that the definition of zoonosis (as defined in the first expert group meeting) had been widely recognized and accepted, and that advocating for the use of “anthropo-zoonoses” and “zoo-anthroponoses” had many drawbacks (9). In addition, this second report also recognized the difference between disease and infection, and slightly modified the definition (Box 1) of zoonoses to be “Those diseases *and infections* which are naturally transmitted between vertebrate animals and man” (9).

**Box 1:** The historical developments in the definition of “zoonoses”. Note that modification(s) adopted if any have been italicized and highlighted.

**Year**	**Zoonosis (Definition)**	**References**
**1950**	**Those diseases which are naturally transmitted between vertebrate animals and man**.	**(8)**
**1958**	**Those diseases *and infections* which are naturally transmitted between vertebrate animals and man**.	**(9)**
**1966**	**No modification(s) suggested**.	**(10)**
**1978**	**Those diseases and infections *[the agents of]* which are naturally transmitted between *[other]* vertebrate animals and man**.	**(11)**

In the 1966 meeting (third report) of the Joint WHO/FAO Expert Group on Zoonoses, the committee recognized that the term zoonosis is etymologically inexact and of little biological merit but found it to be useful enough to promote the prevention and control of zoonotic diseases at the human–animal interface and to provide common ground for medical and veterinary professionals (10). The committee accepted that the definition is too broad and includes situations such as diseases produced by non-infective agents (for example toxins and poisons) and infections that animals acquire from man (merely incidental infections of no public health importance) (10). The wide usage of the term *zoonosis* prevented the Committee/expert group from making any amendments to its definition. However, the Joint WHO/FAO Expert Group on Zoonoses, 1967 (10) did recommend that only those infections with a proof or strong circumstantial evidence of transmission between animals and man should be considered zoonotic diseases.

In 1978, the WHO Expert Committee on Parasitic Zoonoses (with the participation of FAO) agreed with the viewpoint of the Joint WHO/FAO Expert Group on Zoonoses, 1967 (10) and defined zoonoses as “Those diseases and infections [the agents of] which are naturally transmitted between [other] vertebrate animals and man” (11). However, the committee proposed that FAO and WHO should keep this matter under review in light of scientific developments and practical requirements (11). No change in the definition of *zoonosis* has been proposed or discussed since then by the relevant international organizations (WHO, FAO or WOAH).

## 3. Highlighted limitations

Important “definition” issues highlighted by scientists include the absence of clarity on whether to include or exclude zoonotic conditions such as inoculation of vertebrates (humans) by venom or toxins of reptile or fish origin, or by allergens; or diseases transmitted *via* food of animal origin (3, 4). Furthermore, the availability of sufficient evidence that demonstrates natural transmission of many accepted zoonoses has been questioned. There are demands to include unnatural opportunistic infections in immune-compromised patients by organisms of invertebrate origin (12). Unnatural (deliberate) or experimental transmission of human infectious disease agents to other vertebrates is also an issue to consider (12).

It is pertinent to note that the scientific fraternity failed to follow recommendations of the Joint WHO/FAO Expert Group on Zoonoses, 1967 (10) and the WHO Expert Committee on Parasitic Zoonoses with the participation of FAO (11) with respect to differentiating zoonotic and nonzoonotic pathogens.

## 4. Discrepancies in the usage and the confusion thereof

Whether intentional (for the ease of understanding and communication) or not, the World Health Organization has maintained three different versions of the definition of zoonoses:

a) A zoonosis is defined as the disease and infection naturally transmitted between people and vertebrate animals (http://www.emro.who.int/about-who/rc61/zoonotic-diseases.html).

b) A zoonosis is any disease or infection that is naturally transmissible from vertebrate animals to humans (https://www.who.int/news-room/fact-sheets/detail/zoonoses).

c) A zoonosis is an infectious disease that has jumped from a non-human animal to humans (https://www.who.int/news-room/fact-sheets/detail/zoonoses).

A recently published tripartite guide of the international organizations (FAO/WOAH/WHO) also introduces zoonoses as “diseases shared between animals–including livestock, wildlife, and pets–and people” (13).

Furthermore, multiple studies since 1967 classifying or categorizing zoonotic and non-zoonotic pathogens based on different definitions of *zoonoses* have been conducted. Jones et al. (14), Taylor et al. (15), Woolhouse and Gowtage-Sequeria (16), Singh et al. (17), Olival et al. (18) are noted examples. However, there is often a failure to recognize differences in the results produced by such studies due to different methodologic definitions of *zoonosis*. This is a barrier to research in the area of drivers of zoonoses.

Species-jumping is an inherent phenomenon of pathogens. It is believed that most of the novel human pathogens discovered, or yet to be discovered in human populations, are likely to be species-jumping pathogens from other vertebrate animals. There must be a distinction between pathogens that are regularly transmitted from non-human vertebrates to humans (e.g., rabies virus) and those that have jumped from non-human vertebrate(s) to the human population and have become adapted to human-to-human transmission (e.g., HIV, and probably now SARS-CoV-2). If the animal origin was sometime in the past but animals are no longer needed to perpetuate the cycle of transmission, can we still call these zoonoses? It is difficult to think of HIV in the 21^st^ century as a functional zoonotic disease.

This ambiguous and non-specific definition of the term creates problems in classifying diseases. For example, there was a debate in the scientific community at the start of the COVID-19 pandemic about whether to classify COVID-19 as a zoonotic disease. Although the disease is considered to originate from wildlife, COVID-19 virus efficiently transmits between humans and does not require an animal host for maintenance. Similarly, although initially the 2009 H1N1 pandemic influenza jumped from pigs to humans, it did not require any animal species for transmission after it became established in the human population. Would this influenza virus be classified as a zoonotic pathogen? The same also holds true for the human monkeypox virus infections. A consistent and logical classification of zoonotic pathogens is essential when conducting research to characterize these pathogens and explore drivers for the emergence of zoonoses.

## 5. Suggested terminology and classification criteria

The word *zoonosis* is etymologically inexact, but its usage is very common and simple to follow. We suggest that international bodies (WHO/FAO/WOAH) should allow minor modification(s) in the word *zoonosis* and introduce additional terms for differentiating human infections shared among non-human animal and [other] vertebrate species (Table 1).

**Table 1:** Criteria used for the classification of different zoonotic diseases.

**Term**	**Greek usage**	**Transmission type**	**Animals involved**	**Examples**
*Olazoonosis*	óλα (pronunciation “óla,” meaning all)	Natural	Vertebrates and/or invertebrates	Rabies, echinococcosis, malaria and brucellosis
*Akrizoonosis*	ακριβήζ (pronunciation “akrivís,” meaning exact)	Natural	Vertebrates (± invertebrates)	Brucellosis and rabies
*Anakrizoonosis*	ανακριβήζ (pronunciation “anakrivís,” meaning inexact)	Natural	Only invertebrates (No vertebrate)	Non-zoonotic onchocerciasis and malaria
***Akrizoonosis*** **types**				
*Zoizoonosis*	Zoi (pronunciation “zoí,” meaning to live)	Strong circumstantial evidence of an ongoing transmission between vertebrates and humans.	Vertebrates	Brucellosis, rabies, plague, taeniosis and echinococcosis
*Nekrózoonosis*	ν*εκρ*óζ (pronunciation “Nekrós,” meaning dead)	No strong evidence of an ongoing transmission between vertebrates and humans. Human-to-human transmission does not occur or is uncommon.	Vertebrates	*Trypanosoma evansi* infections, foot and mouth disease, and lumpy skin disease.
*Pidózoonosis*	*πηδώντ*αζ (pronunciation “pidóntas,” meaning jumping)	The pathogen jumps from [other] vertebrate species to humans and establishes as anthroponosis. Ongoing human-to- human transmission is very common.	Vertebrates	SARS-CoV-2, Dengue and HIV infections.

We propose the following terminologies to be used for different types of host-based disease categories:

*Olazoonosis*: Those diseases or infections [the agents of] which are naturally transmitted between non-human animals and humans. Note that this term is very broad compared to the existing definition of *zoonosis* and includes infections emanating from both vertebrate and invertebrate species. Note the usage of Greek óλα (pronunciation “óla,” meaning all). Examples include rabies, echinococcosis, malaria, brucellosis and many other transgenerational or transstadial vector borne human diseases. Although infections shared between invertebrate animals and humans do not fit into the scope of the existing definition of *zoonosis*, the WHO uses this broader definition (https://www.who.int/news-room/fact-sheets/detail/zoonoses). The Center for Disease Control and Prevention, USA also defines *zoonotic diseases* (also known as zoonoses) as those caused by germs that spread between animals and people (https://www.cdc.gov/onehealth/basics/zoonotic-diseases.html). In addition, we highlight a paper on emerging infectious disease events published in *Nature* that defined zoonotic pathogens as those that originated in non-human animals (14). However, interpreting and comparing such research with that conducted using the standard definition is unwise. We argue that introducing an additional term *Olazoonosis* will be beneficial for better understanding and differentiating *Akrizoonosis* and *Anakrizoonosis*.

*Akrizoonosis*: Those diseases and infections [the agents of] which are naturally transmitted between [other] vertebrate animals and humans. The definition of *Akrizoonosis* is a synonym of the existing definition of *zoonosis*. Note the usage of Greek ακριβήζ (pronunciation “akrivís,” meaning exact). Examples include rabies, echinococcosis and brucellosis. We believe that introducing the term *Akrizoonosis* would provide options for those tempted to use a broader rather than the standard definition of *Zoonosis*.

*Anakrizoonosis*: Those diseases and infections [the agents of] which are naturally transmitted between invertebrate animals and humans. Note the usage of Greek ανακριβήζ (pronunciation “anakrivís,” meaning inexact). Although human–invertebrate shared diseases do not fit into the existing definition of *Zoonosis*, they are definitely different from human-specific infections (Anthroponosis). *Anakrizoonosis* includes all vector-borne infections such as non-zoonotic onchocerciasis and malaria.

## 6. Classification of zoonoses

We support the classification criteria and different classes of zoonotic infection(s) and disease(s) adopted by the FAO/WHO expert group (10); however, we recommend allowing usage of the etymologically exact *Akrizoonosis* in parallel to the current usage of *zoonosis*.

We also recommend that based on the frequency and temporal trends in the diseases or infections [the agents of], *akrizoonosis* (currently defined *zoonosis*) may be additionally categorized into the following:

1. *Zoizoonosis*: those diseases and infections [the agents of] which are naturally transmissible between [other] vertebrate animals and humans. In addition, there is a proof or strong circumstantial evidence of an ongoing transmission between vertebrates and humans. Note the usage of Greek “Zoi” (zoí; meaning to live). Examples include diseases such as brucellosis, rabies, plague, taeniosis and echinococcosis.
2. *Nekrózoonosis*: those diseases and infections [the agents of] which are naturally transmitted between [other] vertebrate animals and humans. However, there is no strong proof (or only weak circumstantial evidence) of an ongoing transmission between vertebrates and humans. Note the usage of Greek “ν*εκρ*óζ” (Nekrós; meaning dead). This term is intended for those zoonoses which are eradicated or no longer exist in vertebrate animal reservoirs. In addition, diseases with rare zoonotic incidence or presenting with only weak circumstantial evidence–such as *Trypanosoma evansi* infections, and Foot and Mouth disease–could be included within this category of zoonosis. In addition, any disease of debatable or questionable zoonotic potential (for example lumpy skin disease) may also be included.
3. *Pidózoonosis*: those diseases [the agents of] which jump from [other] vertebrate species to humans and establish as anthroponosis (human-specific pathogens). Note the usage of Greek word “πηδώνταζ” (pidóntas; meaning jumping). Examples include dengue, SARS-CoV-2, Dengue and HIV infections.

The Joint WHO/FAO Expert Group on Zoonoses, 1967 recognized that the classification of zoonoses is beneficial, in particular it has value for teaching and that the classification criteria should place emphasis on the epidemiology of zoonotic diseases (10). Similarly, the WHO Expert Committee on Parasitic Zoonoses (with the participation of FAO) noted that there are a large number of zoonotic diseases that demand classification for teaching purposes (11). We believe that the proposed classification perfectly follows the Expert Group guidelines and will enrich and broaden the understanding of Zoonoses by students within medical, veterinary and other related disciplines. It will also highlight the vast differences in the frequency and temporal trends in the transmission of different zoonotic pathogens. The proposed classification will complement the ongoing *reservoir host* and the *type of lifecycle* based classification criteria.

The proposed terms will be valuable for the conduct and understanding of predictive modeling and risk factor investigation studies that use objective zoonotic disease classification data (yes/no) to parameterise different statistical models to determine hotspots or drivers of disease emergence and zoonoses.

Lastly, we would also like to introduce a new term for the infections naturally transmitted among non-human vertebrates.

*Therionosis:* those diseases or infections [the agents of] which are naturally transmitted between nonhuman vertebrate animals. For example, neosporosis. Note the usage of the word “therion” coming from the Greek θ*ηρ* or $\theta \eta \rho \overset{´}{\iota }$*o*ν (meaning wild animal) in this terminology. We believe that identification of such diseases in different animal host species will help develop strategies for comprehensive control of these diseases.

We argue that etymologically exact definitions will be important for clarity and brevity in the future. Although any change in nomenclature will face difficulties in adoption and for understanding of the published literature since 1855, the lack of nomenclature differentiation between diseases naturally transmitted (or transmissible) between nonhuman animals and humans; nonhuman vertebrates and humans; and invertebrates and humans has caused miscommunication and made the scientific literature difficult to interpret for both the scientific and non-scientific communities. It is timely for international bodies (WHO, FAO and WOAH) to reconstitute the Joint WHO/FAO/WOAH Expert Group on Zoonoses and to develop guidelines on the usage of the word “zoonosis.” A focus group discussion in the multidisciplinary One Health High Level Expert Panel (OHHLEP) could also be undertaken. Before being officially introduced, scientific evaluation of the adoption potential of these terms should also be conducted.

## Author contributions

BS: conceptualization, definition(s), and writing—original draft. MW, PK, and ND: manuscript editing and review. All authors contributed to the article and approved the submitted version.
